# Machine learning prediction and classification of behavioral selection in a canine olfactory detection program

**DOI:** 10.1038/s41598-023-39112-7

**Published:** 2023-08-01

**Authors:** Alexander W. Eyre, Isain Zapata, Elizabeth Hare, James A. Serpell, Cynthia M. Otto, Carlos E. Alvarez

**Affiliations:** 1 Center for Clinical and Translational Research, The Abigail Wexner Research Institute at Nationwide Children’s Hospital, Columbus, OH 43205 USA; 2 Department of Biomedical Sciences, Rocky Vista University College of Osteopathic Medicine, Parker, CO 80134 USA; 3 Dog Genetics LLC, Astoria, NY 11102 USA; 4 Penn Vet Working Dog Center, Department of Clinical Sciences and Advanced Medicine, School of Veterinary Medicine, University of Pennsylvania, Philadelphia, PA 19146 USA; 5 Department of Clinical Sciences and Advanced Medicine, School of Veterinary Medicine, University of Pennsylvania, Philadelphia, PA 19104 USA; 6 Departments of Pediatrics and Veterinary Clinical Sciences, The Ohio State University Colleges of Medicine and Veterinary Medicine, Columbus, OH 43210 USA

**Keywords:** Machine learning, Stress and resilience, Social behaviour, Motivation, Behavioural genetics

## Abstract

There is growing interest in canine behavioral research specifically for working dogs. Here we take advantage of a dataset of a Transportation Safety Administration olfactory detection cohort of 628 Labrador Retrievers to perform Machine Learning (ML) prediction and classification studies of behavioral traits and environmental effects. Data were available for four time points over a 12 month foster period after which dogs were accepted into a training program or eliminated. Three supervised ML algorithms had robust performance in correctly predicting which dogs would be accepted into the training program, but poor performance in distinguishing those that were eliminated (~ 25% of the cohort). The 12 month testing time point yielded the best ability to distinguish accepted and eliminated dogs (AUC = 0.68). Classification studies using Principal Components Analysis and Recursive Feature Elimination using Cross-Validation revealed the importance of olfaction and possession-related traits for an airport terminal search and retrieve test, and possession, confidence, and initiative traits for an environmental test. Our findings suggest which tests, environments, behavioral traits, and time course are most important for olfactory detection dog selection. We discuss how this approach can guide further research that encompasses cognitive and emotional, and social and environmental effects.

## Introduction

Machine learning (ML) is a subfield of Artificial Intelligence (AI) that uses a combination of algorithms and statistics to perform a variety of analytical functions on a wide range of data types. ML is split into two algorithm classes: supervised learning for labeled training data, and unsupervised for unlabeled data. Supervised methods allow learning from known inputs and outputs for purposes of prediction of unknown outputs from known inputs (regression analysis), or to determine which data categories are the most important for predicting outcomes (classification analysis). Supervised ML applications in dog behavior have used dog-mounted inertial sensors to create automated dog ethograms sensitive to individual differences^1,2^, and video to classify ADHD-like behavior^3^. Canine unsupervised ML studies have used video and C-BARQ behavioral questionnaire data for exploratory analysis^4^, and sensor data to predict guide dog success^5^. In humans, supervised ML using non-sensor-based, task-relevant testing data has been applied to predicting success in work performance^6^, but we are unaware of such studies in dogs. Here we use supervised methods to predict which dogs will succeed during an odor detection pre-training program or fail for behavioral reasons. While this research has applied use in working dogs, it is also likely to contribute new understandings of learning and work performance in mammals in general, including in humans. However, human behavioral genetics tend to be marked by high levels of heterogeneity, polygenicity, and—due to negative evolutionary selection of even weakly deleterious variations—minute effect sizes of individual variations. Thus, human studies would require vastly greater power and the variations identified would lack direct utility. In contrast, dogs have greatly reduced heterogeneity, polygenicity, and negative selection, and strong positive selection for diverse traits^7^. The ultimate effect is that dogs present dramatically higher power to genetically map all kinds of traits. The downside is that linkage disequilibrium is several-fold more extensive in dogs, resulting in large mapping intervals. However, that can be mitigated by interbreed genetic mapping of variations that are common across breeds^8–10^.

Olfactory detector dogs have long been employed to sniff out explosives, controlled substances, other regulated materials (e.g., insects, food, and plants), and human odor for public safety and security^11,12^. More recently, canine odor detection functions include medical conditions (e.g., low-blood glucose marker in diabetes, and SARS-CoV-2 infection^13^). In the United States, most military and law enforcement dogs are trained as dual-purpose canines, performing both odor detection and protection. The other main groups of working dogs are guide dogs for people with blindness or low vision and service dogs to assist people with other disabilities. The range of costs for most *pre*-trained working dogs is $40,000–80,000^14^, and prices continue to rise because demand exceeds supply. Those costs can be approximately doubled when training is factored in. As a result of those facts and that the overall successful training rate is under 50%, there is a huge impetus to produce and train working dogs more efficiently^11,15^. While there have been exploratory and prospective studies of new testing schemes in detection and assistance working dogs, they have not been deployed widely yet^16^. However, there are large working dog datasets of training, performance and health data from federal and private institutions that have not been thoroughly analyzed yet^15,17^. It thus remains possible that existing standardized datasets, which continue to be collected and are already large and thus ideal for ML, could be the most efficient and productive route to improve the understanding of behavioral traits required for working dogs.

The present work is a study of pre-training success and elimination for behavioral reasons in the Transportation Security Administration (TSA) canine olfactory detection breeding and training program. The data were collected from dogs fostered and tested in the period from 2002 to 2013. During their 15-month fostering period, the dogs were taken to the TSA program facility every 3 months, beginning at the age of 3 months, to be evaluated on a series of tests. The tests evaluated olfaction-dependent traits like the ability to find objects based on odor and other relevant traits such as motivation to possess toys or to play tug of war. At those same times, the handlers also scored the dogs on a variety of other traits, including cooperation with handlers and performance during tasks. At the end of the 12-month testing period, dogs were either accepted into the training program (58.9%) or eliminated for medical (17.2%) or behavioral (23.9%) reasons.

Similar odor detection pre-training and training testing have been used for several decades^18^. The behavioral rating methods used in those have been studied and validated in different ways, including by showing comparable effects of rating and coding approaches in TSA olfactory detection dogs^19–23^. A study similar to ours^20^, of which 106 dogs overlapped our dog population during the same time period, cannot be directly compared to ours because of the many differences. Among those, that study had a priori exclusion criteria that removed dogs likely to be eliminated for behavioral reasons, had sixfold fewer dogs in total, included three breeds vs. one in ours, and the cohort was non-arbitrarily split into two groups of 50% (one used for developing ethograms for behavioral codings and the other for comparing rating vs. coding approaches). A major finding of that work—and the primary question of the study—was to show that the rating methods/data used in that study and ours are comparable to coding methods in predictive validity. That is also important because their coding tests required two- to several-fold more time to perform.

We recently genetically mapped the trait of pre-training elimination for behavioral reasons in the same TSA cohort^17^. The actual reason for elimination in that and the present work is not clearly defined, except that behavioral and medical elimination were distinguished. In the study mentioned above—of 106 dogs of the same working dog population that overlapped the period of ours—the reason for elimination of two dogs was that they “exhibited signs of extreme stress during testing on multiple occasions”^20^. Other behavioral traits that are incompatible with olfactory detection dog selection include poor human or canine socialization, low energy, and elevated levels of excitability, distractibility, aggression, and diverse types of anxiety or fear. Thus, the challenge is that the effects we are trying to identify may be subtle and complex.

In this study, we applied supervised ML algorithms to test how well success or elimination for behavioral reasons can be predicted, and to identify the most important traits at each time or location of testing. Our study of feature classification aims to reveal behavioral test differences that resulted in major temporal or environmental effects on behavioral elimination. This work is part of an ongoing effort to use analytical methods and genomics to improve selection of dogs during their pre-training phase. Our findings suggest developmental and biological effects, and new approaches.

## Results

### 2013 TSA cohort traits

**Figure 1:**
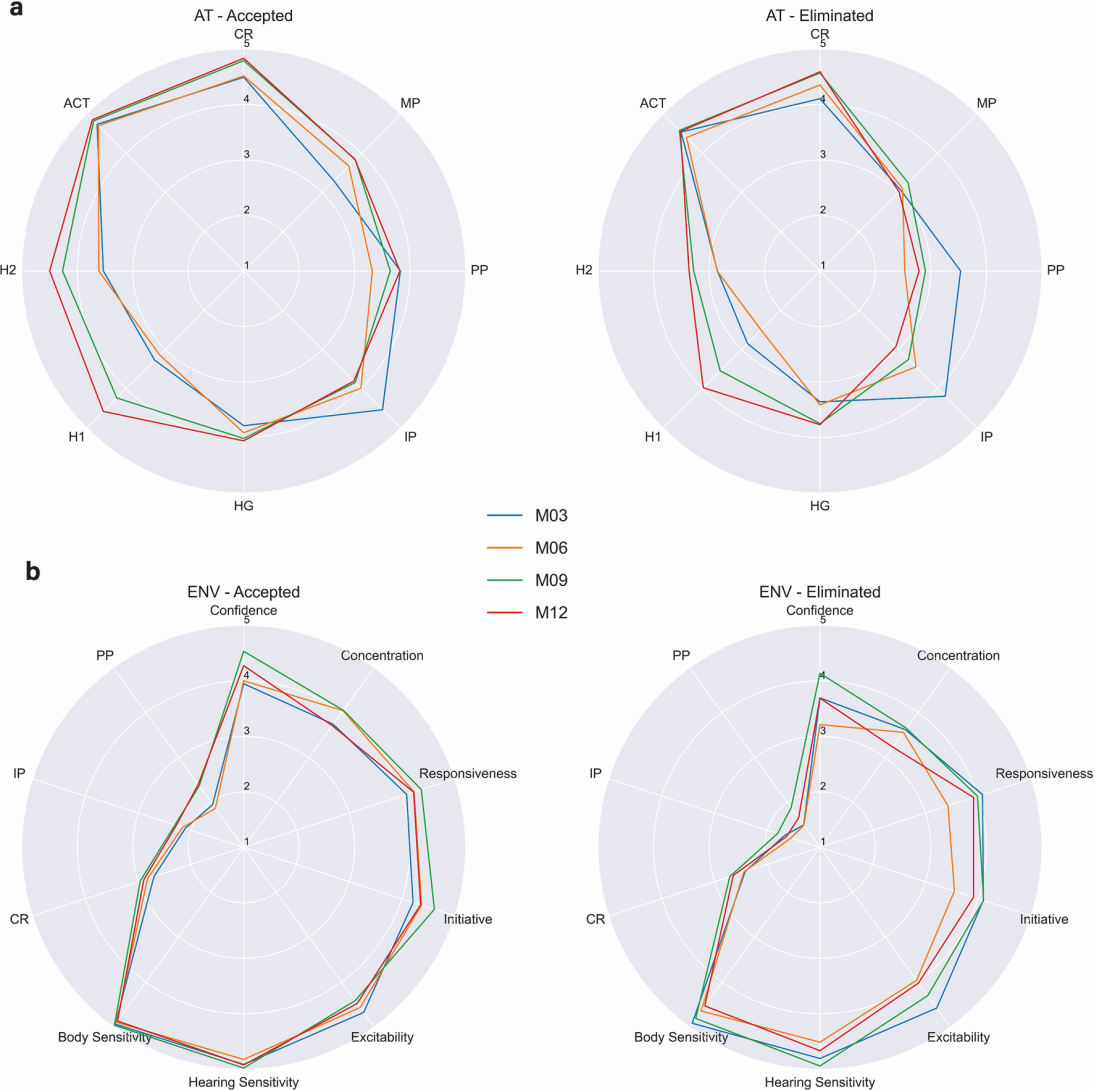
(**a**) Radar plots of the mean scores for each of the traits for the airport terminal tests. (**b**) Radar plots of the mean scores for each of the traits in the environmental tests; M03 = BX (gift shop), M06 = Woodshop, M09 = Airport Cargo, M12 = Airport Terminal.

The traits scored in the cohort represent measures of confidence/fear, quality of hunting related behaviors, and dog-trainer interaction characteristics^19,20^. The traits Chase/Retrieve, Physical Possession, and Independent Possession were measured in both the Airport Terminal and Environmental tests whereas five and seven other traits were specific to each test, respectively (Table 1). The Airport Terminal tests include the search for a scented towel placed in a mock terminal and observation of a dog’s responsiveness to the handler. This represents the actual odor detection work expected of fully trained and deployed dogs. Because the tasks were consistent between the time periods, the Airport Terminal tests demonstrate improvements of the dogs with age. All trait scores except for Physical and Independent Possession increased over time, with the largest increase between the 6- and 9-month tests (Fig. 1a). This may be due to puppies having increased possessiveness and lack of training at younger ages. The general improvement over time could be due to the increased age of the dogs or to the testing experience gained. Compared to accepted dogs, those eliminated from the program for behavioral reasons had lower mean scores across all traits.

**Table 1:** Traits measured by the handlers and the description of what the handlers scored; AT = Airport Terminal, E = Environmental, B = Both.

Trait	Test	Description
MP (Mental Possession)	AT	Ability to focus on a towel, even after being hidden
H1 (Hidden 1)	AT	Concentration, willingness, and ability to move purposefully down a line of upside-down flowerpots, one which contains a hidden scented towel
H2 (Hidden 2)	AT	Second hunt, tester at different location
HG (Hidden Grass)	AT	Enthusiasm and ability to use smell to find a hidden towel
ACT (Activity)	AT	Ability to use his/her energy effectively
CR (Chase/Retrieve)	B	Speed and desire at which the dog runs for a thrown toy
PP (Physical Possession)	B	Desire, force, and determination to play tug-of-war
IP (Independent Possession)	B	Willingness to interact and possess the toy independently of the handler
Confidence	E	Environmentally conditioned acceptance of safety, measure of lack of fear
Concentration	E	Focus during searches, lack of distraction
Responsiveness	E	Ability to react to corrections or encouragement
Initiative	E	Willingness to walk at the end of leash and investigate the environment without being asked
Excitability	E	Enthusiasm during a walk
Hearing Sensitivity	E	Reactivity to noise stimulus during environmental testing
Body Sensitivity	E	Physical reactivity to touch, praise, or correction

Environmental tests involved taking dogs on a walk, a search, and playing with toys in a noisy location that changed for each time point. The traits measured a variety of dog behaviors as they moved through the locations, and their performance while engaging with toys. Accepted dogs had both higher and more consistent scores across the tests (Fig. 1b). The largest separation of scores between accepted dogs and those eliminated for behavior occurred at 6-months, at the Woodshop. That suggests this test and environment combination might best predict which dogs will be accepted into the training program. Among the traits that showed the greatest separation between the two outcomes were Physical and Independent Possession, and Confidence.

### Prediction of pre-training success

Three different classification Machine Learning algorithms were employed to predict acceptance based on their ability to handle binary classifiers: Logistic Regression, Support Vector Machines, and Random Forest. Data were split into training (70%) and testing (30%) datasets with equivalent ratios of success and behavioral elimination status as the parent dataset. Following training of the model, metrics were reported for the quality of the model as described in the Methods. Prediction of success for the Airport Terminal tests yielded consistently high accuracies between 70 and 87% (Table 2). The ability to predict successful dogs improved over time, with the best corresponding to 12-months based on F1 and AUC scores. Notably, this pattern occurred with an overall reduction in both the number of dogs and the ratio of successful to eliminated dogs (Supplemental Table 1). The top performance observed was for the Random Forest model at 12-months: accuracy of 87%, AUC of 0.68, and harmonic mean of recall and precision “F1” of 0.92 and 0.53 for accepted and eliminated dogs, respectively. The Logistic Regression model performed marginally worse at 12-months. Taking the mean of the four time points for accuracy, AUC, and accepted and eliminated F1, Logistic Regression was slightly better than Random Forest for the first three elements and vice versa for the fourth. The Support Vector Machines model had uneven results largely due to poor recall for eliminated dogs (0.09 vs. 0.32 and 0.36 for the other models).

**Table 2:** Metrics for the quality of Machine Learning prediction tasks for the airport terminal (A) and environmental (B) tests.

A				
	M03	M06	M09	M12
Logistic Regression				
Accuracy	0.78	0.76	0.78	0.85
Precision	0.80/0.58	0.77/0.73	0.80/0.58	0.85/0.88
Recall	0.96/0.18	0.97/0.24	0.95/0.22	0.99/0.32
F1	0.87/0.27	0.85/0.36	0.87/0.32	0.91/0.47
AUC	0.571	0.603	0.585	0.653
Support Vector Machine				
Accuracy	0.78	0.74	0.78	0.81
Precision	0.79/0.62	0.74/0.75	0.79/0.67	0.81/1.00
Recall	0.98/0.13	0.98/0.13	0.97/0.19	1.00/0.09
F1	0.87/0.21	0.85/0.22	0.87/0.29	0.89/0.17
AUC	0.553	0.557	0.579	0.545
Random Forest				
Accuracy	0.75	0.70	0.77	0.87
Precision	0.79/0.42	0.75/0.44	0.80/0.53	0.86/1.00
Recall	0.92/0.21	0.87/0.26	0.92/0.28	1.00/0.36
F1	0.85/0.28	0.81/0.33	0.86/0.37	0.92/0.53
AUC	0.561	0.567	0.574	0.681
ML Model	M03	M06	M09	M12

B				
	M03	M06	M09	M12
Logistic Regression				
Accuracy	0.83	0.79	0.80	0.80
Precision	0.84/0.33	0.83/0.46	0.83/0.50	0.82/0.56
Recall	0.98/0.05	0.93/0.24	0.94/0.24	0.94/0.26
F1	0.91/0.09	0.87/0.32	0.88/0.32	0.88/0.36
AUC	0.516	0.584	0.590	0.603
Support Vector Machine				
Accuracy	0.84	0.80	0.81	0.78
Precision	0.84/0.50	0.80/1.00	0.82/0.60	0.78/0.00
Recall	0.99/0.05	1.00/0.04	0.98/0.14	1.00/0.00
F1	0.91/0.10	0.89/0.08	0.89/0.23	0.88/0.00
AUC	0.521	0.520	0.560	0.500
Random Forest				
Accuracy	0.82	0.72	0.78	0.80
Precision	0.84/0.25	0.82/0.30	0.82/0.38	0.81/0.60
Recall	0.97/0.05	0.84/0.28	0.94/0.14	0.97/0.16
F1	0.90/0.09	0.83/0.29	0.87/0.21	0.88/0.25
AUC	0.511	0.558	0.542	0.564

Accuracy is the percentage of correctly identified dogs. Precision is the ratio of true positives to the sum of true and false positives. Recall is the ratio of true positives to the sum of true positives and false negatives. F1 is the harmonic mean of precision and recall. For precision, recall, and F1, the values are reported for accepted/behavioral eliminated dogs. Area Under the Curve (AUC) is the area under the Receiver Operating Characteristics (ROC) curve.

Prediction of success from the Environmental tests yielded worse and more variable results (Table 2). A contributing factor for the poorer performance may have been the smaller mean number of dogs with testing data compared to the Airport Terminal test (56% vs. 73% of the cohort). Overall, the Logistic Regression model was most effective at predicting success based on F1 and AUC scores. That model showed a pattern of improving performance with advancing months. At 12-months, accuracy was 80%, the AUC was 0.60, and F1 were 0.88 and 0.36 for accepted and eliminated dogs, respectively. The best scores, seen at 12-months, coincided with the lowest presence of dogs eliminated for behavioral reasons. Support Vector Machines had extremely low or zero F1 for eliminated dogs at all time points. All three models had their highest accuracy (0.82–0.84) and the highest or second highest F1 for accepted dogs (0.90–0.91) at 3-months. However, all three models had deficient performance in predicting elimination at 3-months (F1 ≤ 0.10).

To maximize predictive performance, a forward sequential predictive analysis was employed with the combined data. This analysis combined data from both the Airport Terminal and Environmental at the 3-month timepoint and ran the three ML models, then added the 6-month timepoint and so on. The analysis was designed to use all available data to determine the earliest timepoint for prediction of a dog’s success (Table 3). Overall, the combined datasets did not perform much better than the individual datasets when considering their F1 and AUC values. The only instances where the combined datasets performed slightly better were M03 RF over the Environmental M03, M03 + M06 + M09 LR over both Environmental and Airport Terminal M09, all data SVM over Airport Terminal M12, and all data LR over Environmental M12. The F1 and AUC scores for the instances where the combined sequential tests did not perform better showed that the ML models were worse at distinguishing successful and eliminated dogs when the datasets were combined.

**Table 3:** Forward Sequential Predictive Analysis for Combined Data. This analysis started with combining both Airport Terminal and Environmental data for M03, then added M06, M09, and M12.

	M03	+ M06	+ M09	+ M12
Logistic Regression				
Accuracy	0.83	0.79	0.84	0.88
Precision	0.84/0.00	0.84/0.00	0.89/0.38	0.91/0.50
Recall	0.98/0.00	0.92/0.00	0.94/0.25	0.97/0.25
F1	0.90/0.00	0.88/0.00	0.91/0.30	0.94/0.33
AUC	0.490	0.462	0.593	0.608
Support Vector Machine				
Accuracy	0.84	0.85	0.87	0.88
Precision	0.84/0.00	0.85/0.00	0.87/0.00	0.91/0.50
Recall	1.00/0.00	1.00/0.00	1.00/0.00	0.97/0.25
F1	0.92/0.00	0.92/0.00	0.93/0.00	0.94/0.33
AUC	0.500	0.500	0.500	0.609
Random Forest				
Accuracy	0.84	0.85	0.87	0.87
Precision	0.86/0.50	0.86/0.50	0.87/0.50	0.87/0.50
Recall	0.98/0.11	0.99/0.06	0.99/0.08	0.99/0.08
F1	0.91/0.18	0.92/0.11	0.93/0.14	0.93/0.14
AUC	0.545	0.526	0.535	0.535

### Feature selection of traits

Two feature selection methods were employed to identify the most important traits for predicting success at each time point: Principal Components Analysis (PCA) and Recursive Feature Elimination using Cross-Validation (RFECV). The PCA was performed on the trait data for each test and no separation was readily apparent between accepted and eliminated dogs in the plot of Principal Components 1 and 2 (PC1/2). Scree plots were generated to show the percent variance explained by each PC, and heatmaps of the top 2 PCs were generated to visualize the impact of the traits within those. Within the heatmaps, the top- or bottom-most traits were those that explained the most variance within the respective component. RFECV was used with Random Forest classification for each test with 250 replicates, identifying at least one feature per replicate. In addition, 2500 replicates of a Naïve Bayes Classifier (NB) and Random Forest Model (RF) were generated to identify instances where RF performed better than a naïve classification.

**Figure 2:**
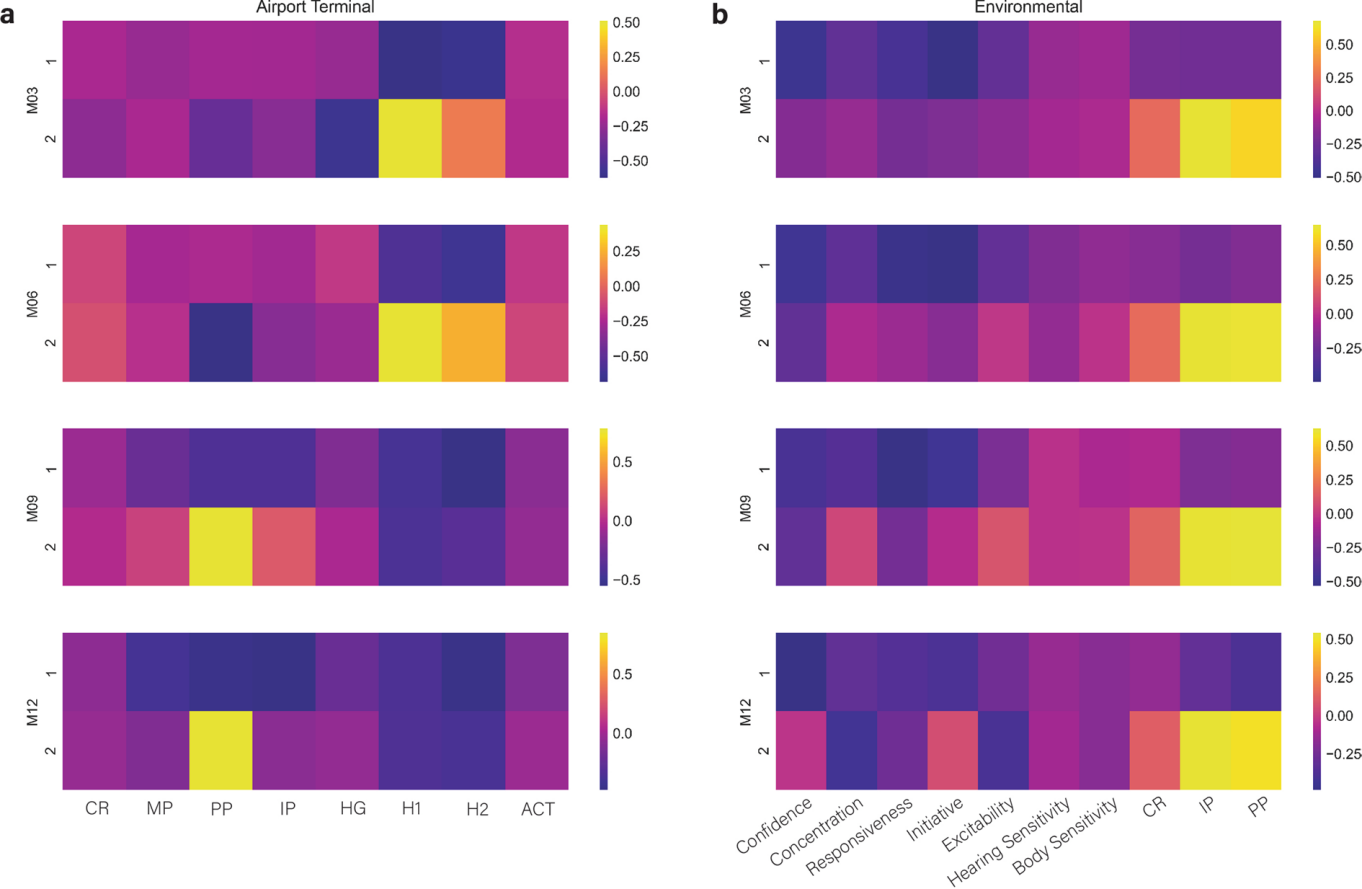
Principal Component Analysis (PCA) results for airport terminal (**a**) and environmental (**b**) tests. Each time point displays a heatmap displaying the relative amount of variance captured by each trait within the top 2 components.

Scree plots of the Airport Terminal tests showed a steep drop at PC2, indicating most of the trait variance is explained by PC1. The variance explained by the top two PCs ranged from 55.2 to 58.2%. The heatmaps (Fig. 2a) showed the PC1/2 vectors with the strongest effects were H1/2 at 3- and 6- months, and PP at 9- and 12-months, both of which appeared in the upper left quadrant (i.e., negative in PC1 and positive in PC2). Several traits showed temporal effects within PCs: (i) at 3-months, PC1 had lower H1 than H2 scores, but that reversed and its effect increased at the other time points; (ii) at 3- and 6-months, PC2 had positive signal for H1/2, but both became negative at 9- and 12-months; (iii) at 3-months, HG was negative, but that effect was absent at other time points; (iv) at 3- and 6- months, PC2 had negative signal for PP, but it changed to strongly positive at 9- and 12-months. When the RFECV was run on the same Airport Test data, a similar pattern of increasing number of selected traits with advancing time points was observed as in the PCA (Table 4). Like the PCA results, H2 was among the strongest at all time points except for the 6-month, although it first appeared among the replicates at 9-months. Means of the NB and RF models were compared (Supplemental Table 2) and showed the M06 and M12 results were the most promising for classification. This suggested that shared traits such as all possession traits (MP, IP, and PP) and the second hunt test (H2) are the most important in identifying successful dogs during these tests, however the distinct nature of the assessment in each time point does not allow for a longitudinal interpretation.

**Table 4:** Recursive Feature Elimination with Cross-Validation using Random Forest Classification results for airport terminal (A) and environmental (B) tests.

	MP	H1	H2	HG	ACT	CR	PP	IP		
A										
3 MO	4.7	–	30.7	86.7	–	–	–	–		
6 MO	24.7	–	10.7	–	–	–	100.0	47.3		
9 MO	–	12.7	53.3	–	–	–	64.0	19.3		
12 MO	48.7	38.7	77.3	28.7	0.0	6.0	51.3	76.0		

	Conf	Conc	Respon	Init	Excit	Hear Sens	Body Sens	CR	PP	IP
B										
3 MO	100.0	–	–	0.4	–	–	–	–	–	–
6 MO	88.7	–	4.7	13.3	–	–	–	–	–	–
9 MO	20.7	16.0	23.3	30.7	16.7	0.8	12.0	14.0	23.3	93.3
12 MO	63.3	48.9	32.0	32.7	46.7	21.3	22.7	28.7	80.7	47.3

Values indicate the percentage of 250 bootstrap runs the respective traits appeared in, ranging from 0 to 100.

The PCA results for the Environmental tests yielded scree plots that had a sharp drop at PC2 for all time points except 9-months (Fig. 2b). The amount of variation explained by the top two components decreased with the increasing time points from 62.7 to 49.8. The heatmaps showed the PC1/2 vector with the strongest effect was for the toy possession trait IP, which appeared in the upper left quadrant at all time points (CR and PP had a similar effect at reduced magnitudes). Within PC observations included the following: (i) in PC1, Confidence and Initiative were negative at all time points, and (ii) in PC2, Concentration and Excitability were positive at 3-months, and increased at 6- and at 9- and 12-months. When the RFECV was run on the Environmental test scores (Table 4), all traits for both 9- and 12- months were represented in the results. At 3-months, only Confidence and Initiative were represented and at 6-months, only those and Responsiveness. Means of the NB and RF models were also compared (Supplemental Table 2) and demonstrated M03 and M12 were the most significant for classification. These tests correspond to the earliest test at the gift shop and the last test at an active airport terminal. Primary shared traits include confidence and initiative, with possession-related and concentration traits being most important at the latest time point.

## Discussion

This exploratory study tested the feasibility of using supervised Machine Learning approaches to extract useful knowledge from an existing, large dataset of pretraining tests, behavioral traits, and environmental contexts for Labrador Retriever working dogs. We used 70% of the dogs for learning and 30% to test the prediction of which dogs were successful in a pre-training program or eliminated for behavioral reasons. We saw the best performance for the Random Forest model at 12-months in the Airport Terminal test, with accuracy of 87% and an AUC—the ability to distinguish between accepted and eliminated dogs—of 0.68. In general, AUCs of 0.5–0.7 are considered poor and 0.7–0.8 acceptable. The weakest metric for that model, test, and time point was for recall—the ability to find all positive instances—for eliminated dogs of 0.36 (vs. 1.0 for accepted dogs). This resulted in an F1—the harmonic mean of recall and precision—of 0.53 (vs. 0.92 for accepted dogs). One consideration for this result is that the Airport Terminal test had a mean of 73% of the total 628 dogs with data across all traits (and only 56% for the Environmental test). A second factor is the breeding selection exerted on our cohort and the broader Labrador Retriever populations it was derived from.

We previously reported genetic mapping of elimination for behavioral reasons in the same cohort^17^. There we referenced the behavioral selection related to this cohort and the general population of “hunting line” Labrador Retrievers. The findings in that work showed variations that are associated with problem behaviors, and which are common in pet Labrador Retrievers, are rare or absent in the present cohort. For instance, an X chromosome allele associated with fear, anxiety, and aggression, (likely due to a coding variant in *IGSF1*^8,10^) has an allele frequency of 18% in pet Labrador Retrievers but was not detected in ~ 300 dogs in this cohort. Similarly, our mapped haplotypes with strongest effects on elimination for behavioral reasons tended to only be present in the heterozygous state. In this way, breeding selection results in depletion of alleles associated with moderate to large-effect problem behaviors observed in the general pet population; therefore, a reduction of both behavioral variance and rates of elimination makes their discovery more challenging in specialized cohorts^8–10^.

The predictive performances of the models for the Environmental test were more variable and poorer. The accuracy for the top performing Logistic Regression model was 80% at 12-months. The AUC was 0.60 and the F1s 0.88/0.36 for accepted and eliminated dogs, respectively (mainly resulting from recall rates of 0.94/0.26). An important caveat for the Logistic Regression model is that some traits exhibited a biased distribution toward higher scoring values, which may bias the reported metrics. The pattern of metrics was different in the two tests. In the Airport Terminal test, all top metrics were for Random Forest at 12-months, and all second-best for Logistic Regression at 12-months. In contrast, the Environmental test had the top or second highest metrics for accuracy and accepted dog precision and recall (and thus F1) at 3-months. However, all three models had a recall rate for eliminated dogs of 0.05 (and F1 of 0.09–0.10). This suggests the dogs most likely to be accepted (~ 60% of cohort) can be recognized through features in our data for 3-months. That is not the case for identification of dogs likely to be eliminated (~ 25% of the cohort; the remainder eliminated for medical reasons). Since the recall rate of eliminated dogs is over five-fold higher at later time points for both the Logistic Regression and Random Forest models, it may be possible to determine the developmental timing of the traits responsible for pre-training success.

The PCA of the Environmental test was less variable across time points than the Airport Terminal test. This seems surprising given the more variable results observed in the predictive modeling of the Environmental test. It is also unexpected considering the Environmental test was given at different types of location chosen to present different types of stimuli. The first two PCs explained a decreasing proportion of the variance with advancing time points, from 62.7 to 49.8%. The strongest effect present for the PC1/2 combination was for the toy possession trait IP, which plots to the top left quadrant at all time points. Chase/Retrieve and the PP had similar but smaller effects. Single PC observations included that Confidence and Initiative were moderately to strongly negative in PC1 at all time points. Among the temporal effects in PC2, Concentration and Excitability were weakly positive at 3-months and increased slightly at 6- and again at 9- and 12-months. Running the RFECV showed all traits at 9- and 12-months were positive in the results. Confidence and Initiative were represented at all time points, and Confidence had the most consistently high classification values (100, 88.7, 20.7, and 63.3% in order of increasing time points. At 3- and 6-months, Confidence was highest (100 and 88.7%); at 9-months, IP was (93.3%); and at 12-months, PP was (80.7%).

Overall, our Machine Learning algorithms were not effective in predicting success during a explosives-detection pretraining program. They also displayed a poor ability to properly distinguish between the successful dogs and those eliminated, rendering the application of these models unsuitable for unsupervised use. This may be due in part to our data lacking thorough documentation of the basis for graduating or removing dogs. This could help explain the improved performance of the algorithm over time; however, the separation could also be due to the dogs’ behavioral development and learning. While the AUC scores were not strong, the classification results shed light on the most robust traits that are important for success. These results are consistent with previous studies that primarily used PCA and Factor Analysis to identify important traits in an overlapping cohort that contained multiple breeds and combined all time points in some cases^22^. Those studies demonstrated that Responsiveness, Initiative, Confidence, and Concentration (with PC1 loading values of 0.92, 0.86, 0.81, and 0.67, respectively) contributed the most to dog success in the Environmental Tests. Also, Mental Possession, Independent Possession, Hidden 1, Hidden 2, and Physical Possession (with PC1 loading values of 0.74, 0.66, 0.64, 0.60, and 0.55) contributed the most to dog success in the Airport Terminal test^20,22^. Those results were consistent with both our PCA and ML Classification tasks. Another study showed a similar phenomenon of shifts in the consistencies of scores (e.g., with environmental sureness and possession-related traits) between time points^23^ using PCA. This trend may capture the development of dog behavior as they age from 3 to 12 months of age, although this likely also captures experience with the tasks and some change due to the bit of training expected of handlers during this pre-training period.

### Conclusions

This study provided a preliminary look into the predictive power of ML algorithms to select successful Labrador Retrievers in a canine olfactory detection pretraining program. The results demonstrated a subset of the traits that may be more important than the others for the selection of successful dogs, which has the potential to simplify trait assessments in the program. While the ability to distinguish between successful and behaviorally eliminated dogs was poor, our data only represent a small cohort of dogs with few traits. Our findings indicate there are great opportunities to expand upon the program by including additional behavioral traits, medical information, and other longitudinal data.

## Materials and methods

### 2002–2013 TSA cohort and data

Data for the study was obtained from an olfactory detection dog breeding and training program run by the TSA in the period from 2002 to 2013. This data contained scores for 628 Labrador Retrievers that were brought in for testing every 3 months beginning at the age of 3 months during a 15 month foster period. These testing periods correspond to a 3-, 6-, 9-, and 12-month time period when two separate tests were performed. The first test, called the Airport Terminal (AT) test, was performed in an empty mock airport terminal and was meant to simulate the intensive training the dogs would perform if they passed the pre-training program. This test involved the handlers walking the dogs through the mock airport terminal, two separate hunts for a scented towel in vessels scattered throughout the terminal, and engagement with a toy. The traits measured the dogs’ performance while identifying the scented towel, qualities of the dog during the tasks, and level of engagement with the handler, towel, and toy. This test was meant to demonstrate how trainable the dog would be if it were successful.

The second test, called the Environmental (Env) test, was performed in different locations around the base at each time point. The test involved the dog walking with the handlers on a leash, attempting a search, and engagement with a toy and the handler while in a noisy and crowded environment. The locations included a busy base exchange gift shop (BX), a woodshop with loud noises and dark enclosed spaces (Woodshop), a cargo area with moving traffic and noise (Airport Cargo), and various airport passenger locations (Airport Terminal), respectively to the four time points. This test complemented the airport terminal test as there were no other people in the mock airport terminal to distract the dogs from the task at hand. The Environmental test captured traits that measured various characteristics of the dogs when in these stimulating locations and their ability to still focus on the various aspects of training.

Of the 628 dogs included, a fraction was scored at each time point. That ranged from 351 to 564 for the Airport Terminal tests and 291 to 410 for the Environmental Tests. All the dogs had accepted or eliminated status for medical or behavioral reasons (otherwise unspecified), and their overall counts are summarized in Supplemental Table 1. Dogs eliminated for medical reasons were included in this behavioral study as their medical conditions were not described, and for those that were found, were mainly issues that would limit a dog’s longevity in the program (eg. Hip dysplasia) and not necessarily effect behavior.

### Data preparation and visualization

Data for the dogs were split based upon the type of test and time period, and dogs with substantial (> 25%) missing scores were dropped for those tests. The distributions of trait scores were visualized by first splitting the datasets based on whether the dogs were accepted or eliminated for behavioral reasons (see code). The mean score for each trait was calculated and plotted on a radar plot using *matplotlib v3.4.2* and *plotly v5.3.1*, packages of *Python 3.8.12*. Data and Jupyter Notebook code are available at https://github.com/AWEyre7147/2013TSA-Trait-ML-Project.

### Machine learning prediction and classification

All Machine Learning was performed using the corresponding toolkits in *scikit-learn v0.24.2* with random state of 101 unless otherwise noted^24^. Predictive Machine Learning models were selected for their ability to handle binary classifiers and unique means of making predictions. For predictive tasks, the data were split into training/test sets using a test size of 30%. A Logistic Regression model was run using default settings. A Support Vector Machine model was run using default settings, then an attempt to refine the model was performed using a grid search with a range of *C* and *gamma* values (see code). A Random Forest model was run using 100 estimators. Quality of all models was assessed using classification reports and calculation of the AUC statistic. Accuracy is the percentage of correctly classified dogs ((true positives + true negatives)/(true positives + false negatives + true negatives + false positives). Recall is the is the ability of a classifier to find all positive instances (true positives / (false negatives + true positives)). Precision is the proportion of positives predictions that are correct (true positives/(false positives + true positives)). F1 is the harmonic mean of recall and precision (F1 Score = (2 * Precision Score * Recall Score)/(Precision Score + Recall Score)). The receiver operating characteristics (ROC) curve was also created, and the AUC was calculated, which is the ability of a model to distinguish between positive and negative classes.

For classification Machine Learning tasks, principal components analysis (PCA) was performed, and scree plots were generated to visualize the percentage of variance explained by the components. The first two components were selected because they lie before the inflection point of the scree plot curve; and a heatmap was generated to visualize which traits most impacted each component. To identify which features are most important, recursive feature elimination with cross-validation (RFECV) was performed using a random forest classifier model. RFECV chooses the optimal number of features by using cross-validation (CV). We employed this method to demonstrate that the number and importance of each trait increases as the dogs become more trained. It was run searching for a minimum of 1 feature and replacement scoring based on accuracy, then bootstrapping was run 250 times with random states ranging from 1 to 250. Traits that were selected after each run were collected, then the % of runs each trait occurred in was reported for each test and time period. To provide a baseline to compare the RF predictive accuracies and provide validity to the RFECV results, 250 replicate Naïve Bayes Classifier and Random Forest runs were calculated for each time point and test with the mean and standard deviation reported with Z-tests calculated for each pair.

## Supplementary Information

Supplementary Information.

## Data Availability

Data and computer code are available at https://github.com/AWEyre7147/2013TSA-Trait-ML-Project.
