# Supplementary material for: Machine learning prediction and classification of behavioral selection in a canine olfactory detection program
Source: Sci Rep. 2023 Aug 1;13:12489. doi: 10.1038/s41598-023-39112-7 (PMC10394074; doi:10.1038/s41598-023-39112-7)
Supplement: Supplementary file 1 — Supplementary Information. [file 41598_2023_39112_MOESM1_ESM.docx]

**Supplementary tables**

|  | M03 | M06 | M09 | M12 |
| --- | --- | --- | --- | --- |
| **A.T. Count** | 564 (89.8) | 545 (89.8) | 445 (70.9) | 351 (55.9) |
| Accepted | 327 (52.1) | 406 (64.6) | 344 (54.8) | 290 (46.2) |
| Eliminated | 142 (22.6) | 139 (22.1) | 101 (16.1) | 61 (9.7) |
| Missing Acpt. | 154 (24.5) | 75 (11.9) | 137 (21.8) | 191 (30.4) |
| Missing Elim. | 5 (0.8) | 8 (1.3) | 46 (7.3) | 86 (13.7) |
| **Env Count** | 389 (61.9) | 410 (65.3) | 352 (56.1) | 291 (46.3) |
| Accepted | 327 (52.1) | 328 (52.2) | 283 (45.1) | 246 (39.2) |
| Eliminated | 62 (9.9) | 82 (13.1) | 69 (11.0) | 45 (7.2) |
| Missing Acpt. | 154 (24.5) | 153 (24.4) | 198 (31.5) | 235 (37.4) |
| Missing Elim. | 85 (13.5) | 65 (10.4) | 78 (12.4) | 102 (16.2) |

**Supplemental Table 1.** Miscellaneous data related to the counts of the Labrador Retrievers in each of the tests and categories. Parentheses contain the % of total dogs.

**A.**

|  | M03 | M06** | M09 | M12*** |
| --- | --- | --- | --- | --- |
| Naïve Bayes | 0.73±0.03 | 0.73±0.03 | 0.76±0.03 | 0.80±0.03 |
| Random Forest | 0.73±0.03 | 0.74±0.03 | 0.75±0.03 | 0.83±0.03 |

**B.**

|  | M03*** | M06 | M09 | M12*** |
| --- | --- | --- | --- | --- |
| Naïve Bayes | 0.76±0.10 | 0.78±0.04 | 0.79±0.08 | 0.79±0.04 |
| Random Forest | 0.81±0.03 | 0.78±0.03 | 0.79±0.03 | 0.83±0.03 |

**Supplemental Table 2.** Gaussian Naïve Bayes Classification for airport terminal (**A**) and environmental (**B**) tests, and its statistical comparison to the accuracies of the Random Forest model. Naïve Bayes and Random Forest accuracies are reported as the mean of 250 runs of 70/30% training/test split ± standard deviation. Z-test significance * < 0.05; ** < 0.005; *** <0.0005
